# Supplementary material for: Collective navigation of complex networks: Participatory greedy routing
Source: Sci Rep. 2017 Jun 6;7:2897. doi: 10.1038/s41598-017-02910-x (PMC5460226; doi:10.1038/s41598-017-02910-x)
Supplement: Supplementary file 5 — Supplementary Materials [file 41598_2017_2910_MOESM5_ESM.pdf]

Supplementary Materials for "Collective navigation of complex networks: Participatory greedy routing"

Kaj-Kolja Kleineberg[1,*] and Dirk Helbing[1]
[1] *Computational Social Science, ETH Zurich, Clausiusstrasse 50, CH-8092 Zurich, Switzerland*
(Dated: March 9, 2017)

CONTENTS

I. Explicit realizations 1
II. Bistability 3
III. Performance of greedy routing and participatory greedy routing 6
IV. The impact of heterogeneity 6
V. Supplementary Videos 6

I. EXPLICIT REALIZATIONS

In Fig. 1 we show explicit realizations of the system.

* kkleineberg@ethz.ch

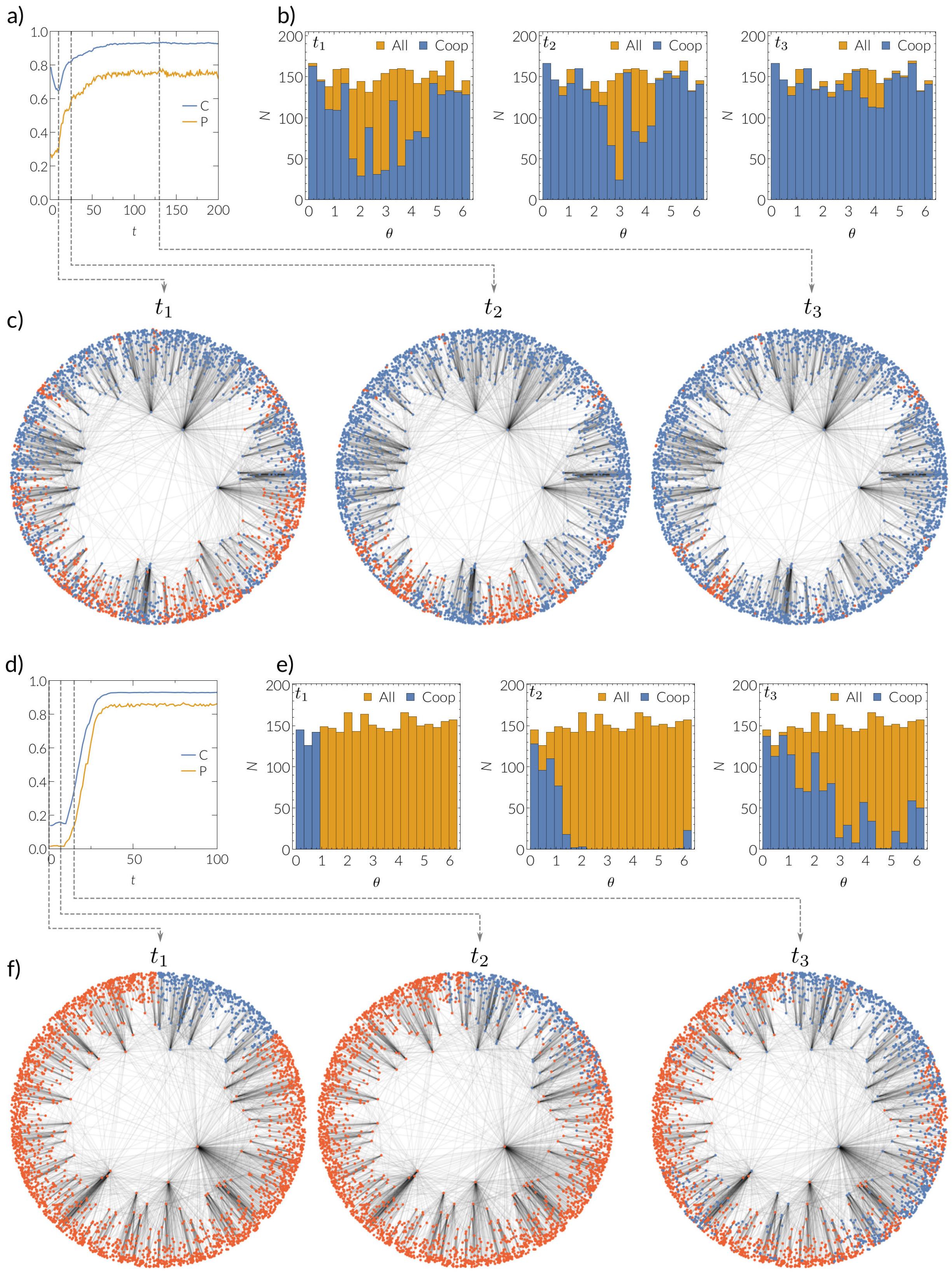

Figure 1. **a)** Emergence of cooperation in a network for $b = 10$ and $C_0 = 0.8$ with $N = 3000$ nodes, power law degree distribution with exponent $\gamma = 2.5$, temperature $T = 0.4$, and mean degree $\langle k \rangle \approx 6$. Initial cooperators are distributed randomly. We show the evolution of the density of cooperators (blue line) and the success rate of navigation (yellow line). Time t represents the number of update phases the system has undergone. **b)** shows histograms for the same realization for the number of cooperators (blue) and total number of nodes (yellow) in respective bins of the angular coordinate θ. Time increases from left to right, $t = (10, 25, 130)$. **c)** shows for the same realization the network and the states of the nodes for times $t = (10, 25, 130)$. See Supplementary Video 3. **e-f)** The same as **a-c** but for $C_0 = 0.15$, $b = 25$, and localized initial conditions. Times shown are $t = (0, 7, 15)$. See Supplementary Video 4.

II. BISTABILITY

In Figs. 2 and 3 we show histograms of the density of cooperators in the stationary state for synthetic networks. Parameters are the same as in the main paper in Fig. 2a. We note that only two qualitatively different final states are possible: one with very high cooperation and one with nearly complete defection. To compute the quantities in the stationary state, we first let 250 rounds each consisting of N message sending events and an update phase pass, which avoids the initial transient phase, and then we average over another 250 rounds. We confirm that indeed only two qualitatively opposite states are approached by the system. We find the same behavior using the real Internet topology instead of synthetic networks.

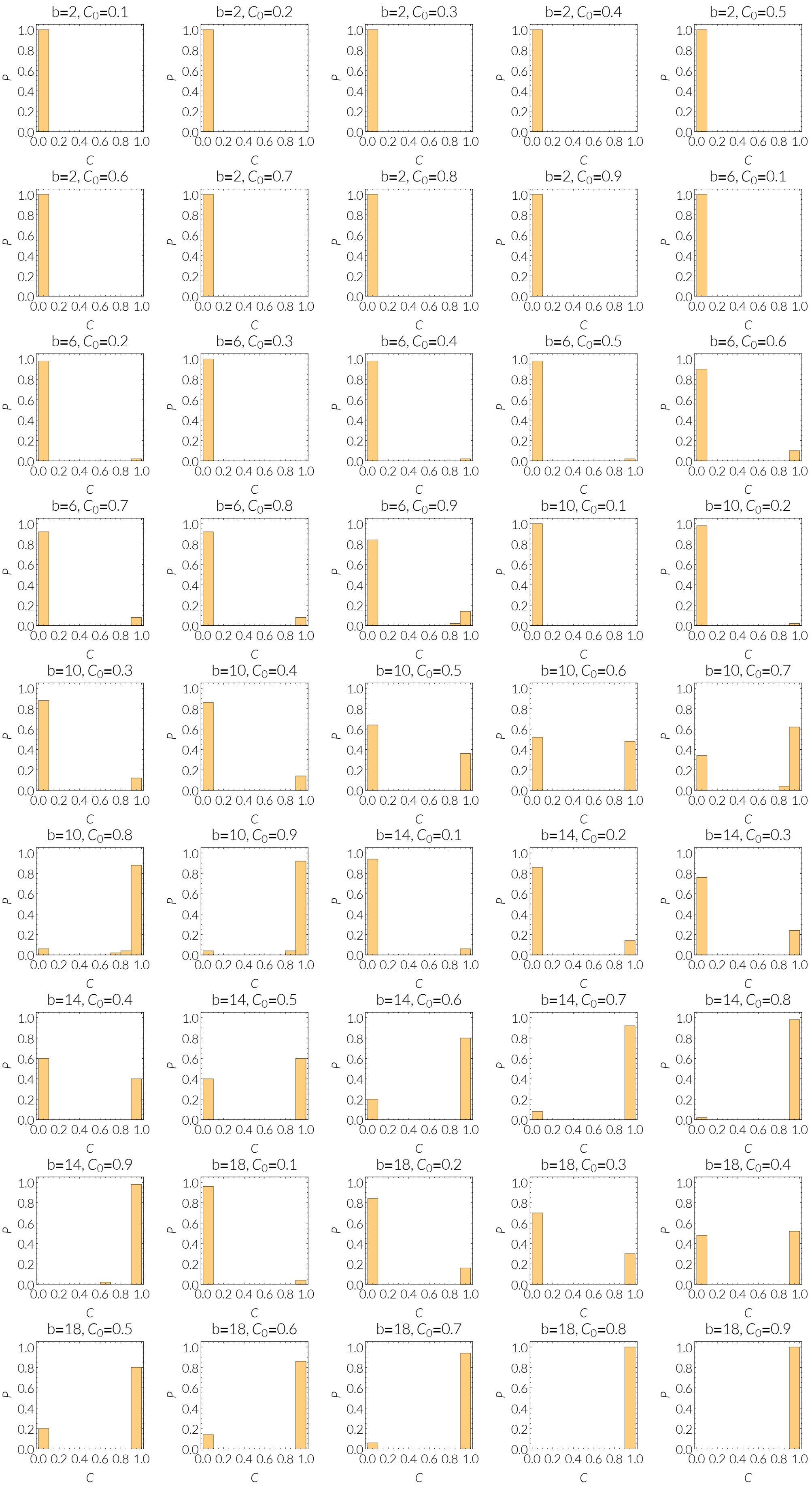

Figure 2. Distribution of the density of cooperators in the stationary state among 50 realizations of our model using synthetic networks and the parameters as in the main paper in Fig. 2a. We observe the two distinct final states: very high or very low cooperation and system performance respectively. Note that for low payoff parameters, clusters of defectors can persist, as shown in Fig. 1 for an explicit realization. Therefore, in some cases we observe final cooperation densities of approx. 80% (see for instance $b = 10$ and $C_0 = 0.8$).

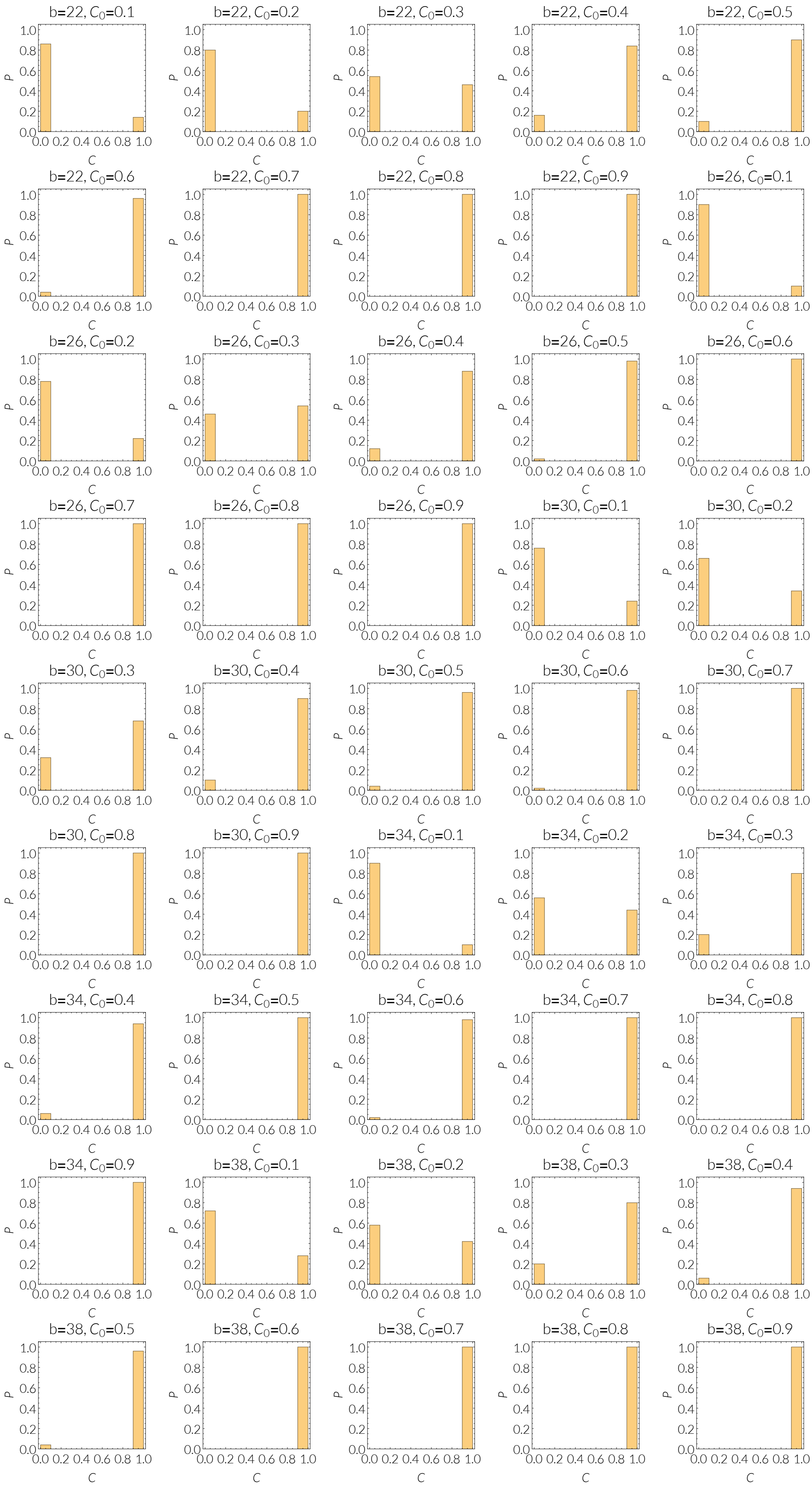

Figure 3. Continuation of Fig. 2.

III. PERFORMANCE OF GREEDY ROUTING AND PARTICIPATORY GREEDY ROUTING

In Fig. 4 we show the performance of greedy routing and participatory greedy routing as a function of the power law exponent γ. Note that the navigability of the system, even if every agent cooperates (GR), decreases quickly for values of $\gamma \gtrsim 2.9$.

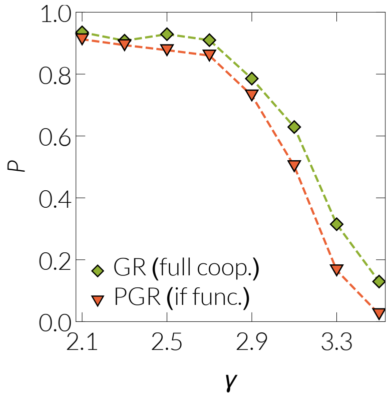

Figure 4. Performance of greedy routing (GR) and participatory greedy routing (PGR) (if the system is functional). For PGR, show the average success rate the system approaches for $C_0 = 0.9$ and $b = 35$. Networks parameters are as in the main text in Fig. 2c.

IV. THE IMPACT OF HETEROGENEITY

Figs. 5 and 6 show results as in the main paper Fig. 2 for different values of γ.

V. SUPPLEMENTARY VIDEOS

This work contains four Supplementary Videos, for which the captions are provided below.

- Supplementary Video 1:
  Emergence of cooperation in a synthetic network for $b = 25$ and $C_0 = 0.15$ with $N = 3000$ nodes and a power law degree distribution with exponent $\gamma = 2.5$, temperature $T = 0.4$, and mean degree $\langle k \rangle \approx 6$. We show the evolution of the density of cooperators (blue line) and the success rate of navigation (yellow line). Time t represents the number of update phases the system has undergone. Histograms show the number of cooperators (blue) and total number of nodes (yellow) in respective bins of the angular coordinate θ.

- Supplementary Video 2:
  Same as Supplementary Video 1, but for the IPv6 Internet network and for $C_0 = 0.15$ and $b = 30$.

- Supplementary Video 3:
  Emergence of cooperation in a network for $b = 10$ and $C_0 = 0.8$ with $N = 3000$ nodes, power law degree distribution with exponent $\gamma = 2.5$, temperature $T = 0.4$, and mean degree $\langle k \rangle \approx 6$. Initial cooperators are distributed randomly. We show the evolution of the density of cooperators (blue line) and the success rate of navigation (yellow line). Time t represents the number of update phases the system has undergone. Histograms show the number of cooperators (blue) and total number of nodes (yellow) in respective bins of the angular coordinate θ.

- Supplementary Video 4:
  The same as Supplementary Video 3 but for $C_0 = 0.15$, $b = 25$, and localized initial conditions.

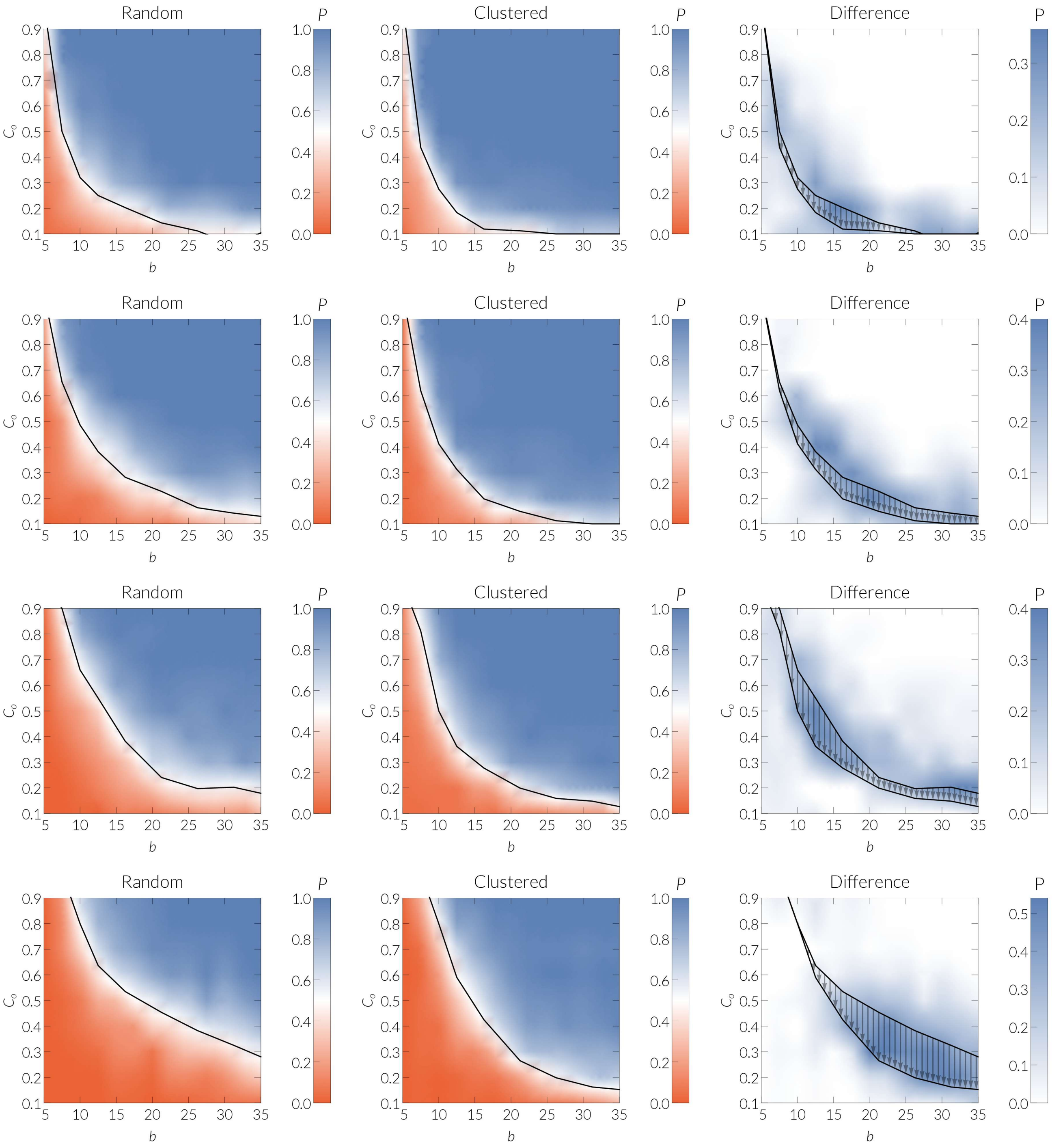

Figure 5. Results as in the main paper in Fig. 2 for different values of γ. From top to bottom: $\gamma = (2.1, 2.3, 2.5, 2.7)$. First column shows the probability to reach the functional state for random initial conditions, the second row shows the same but for clustered initial conditions. The third row shows the difference between these probabilities, i.e. $P_{\text{clustered}} - P_{\text{random}}$. Black lines denote the threshold value of C_0 above which the functional state is reached with more than 50% probability.

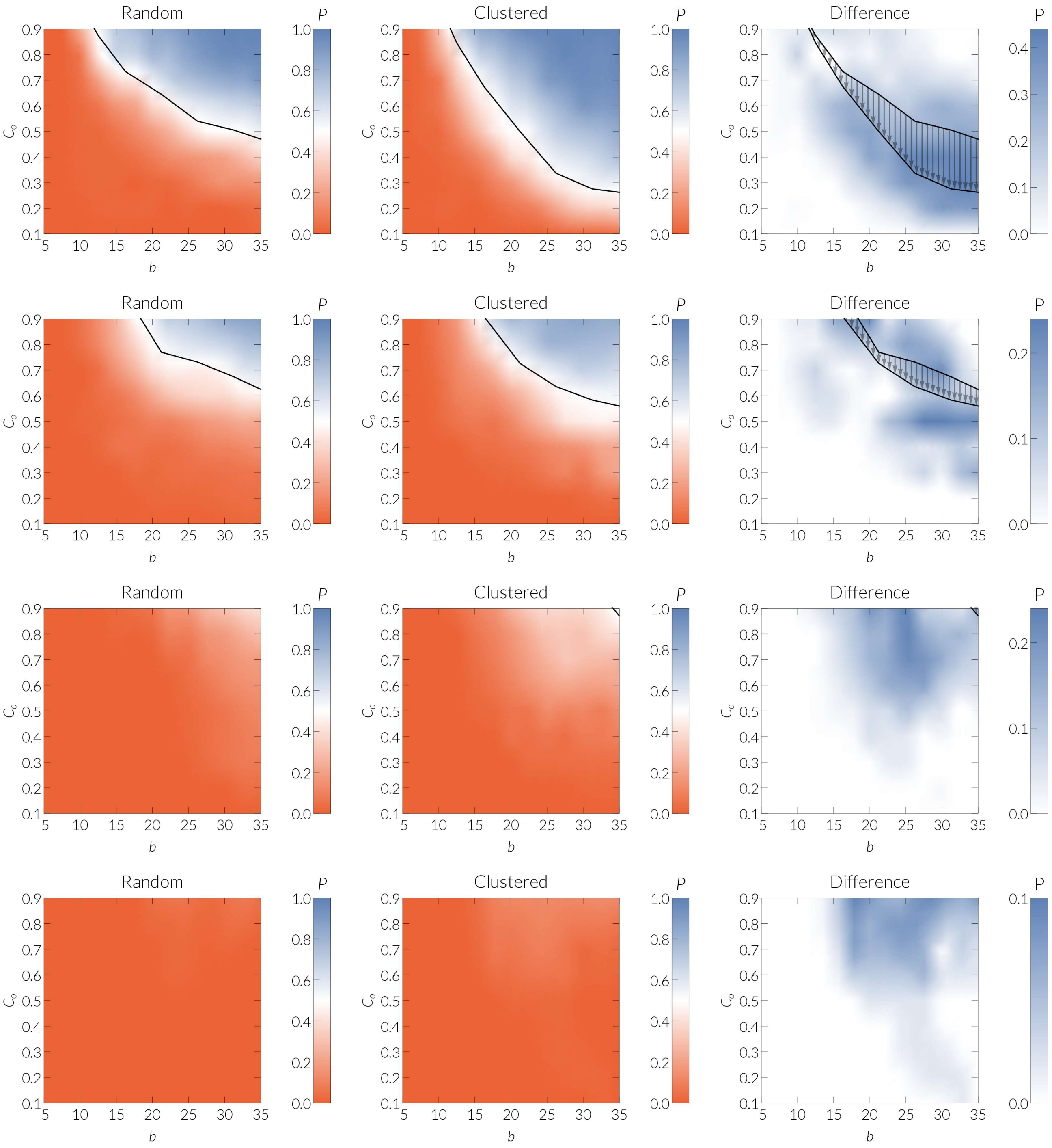

Figure 6. As in Fig. 5 but for $\gamma = (2.9, 3.1, 3.3, 3.5)$ from top to bottom.
